# Remarkable anecdotes illustrating the nature and effect of seizure-precipitating factors in Border Collies with idiopathic epilepsy

**DOI:** 10.3389/fvets.2023.1254279

**Published:** 2023-09-14

**Authors:** Paul J. J. Mandigers, Koen M. Santifort

**Affiliations:** ^1^Evidensia Referral Hospital Arnhem, Arnhem, Netherlands; ^2^Expertise Centre of Genetics, Department of Clinical Sciences, Faculty of Veterinary Medicine, Utrecht University, Utrecht, Netherlands

**Keywords:** idiopathic epilepsy, dog, epileptic seizure, triggers, hereditary

## Abstract

Epilepsy is one of the most common chronic neurological syndromes in dogs and has serious implications for the quality of life of both the dogs and owners. Seizure-precipitating factors (SPFs) (also termed “triggers” or “provocative factors”) have been studied and reported in both humans and dogs with idiopathic epilepsy. In dogs stress, hormones, sleep deprivation, and the weather have been reported as SPFs. The Border Collie (BC) is a breed of dog that is predisposed to idiopathic epilepsy, and the outcome is often poor. BC is described as a very sensitive dog with a strong focus on their owners, and this may have an influence on their and their owners' stress level. In this article, we described six unrelated BCs with idiopathic epilepsy in which several remarkable SPFs were identified, and avoiding them improved the outcome of these dogs. The possible SPFs were different for each dog. The SPFs were, among others, the other dog in the family, the lack of intellectual challenge, the presence of an autistic child, a busy street, the relation with the owner, and throwing a ball at the beach. These cases illustrate that recognizing the SPF(s) and taking measures with regard to management can lead to a reduction in epileptic seizure frequency or even achieving seizure freedom.

## Introduction

Epilepsy is one of the most common chronic neurological syndromes in dogs (1) and has serious implications on the quality of life (QoL) of both the dogs and owners (2–6). Regarding QoL, as assessed by owners of dogs with idiopathic epilepsy (IE), a recent study of our group demonstrated clear breed-related differences (6). QoL of both owner and dog was scored significantly lower by owners of Border Collies (BCs) compared with owners of other dog breeds, such as Golden Retriever or Labrador Retriever (6). The frequency of epileptic seizures (ES) and cluster seizures (CS) in Golden Retrievers and Labrador Retrievers was markedly lower than in BCs (6). The semiology in these three breeds is comparable (7), but the outcome is noticeably different. Half of all male BCs with IE that develop ES at 1 to 2 years of age die or are euthanized due to the lack of adequate seizure control (6, 8). As a genetic factor is suspected in numerous breeds with a high prevalence of IE (7), genetic studies have been performed in a number of breeds (9–12), including the BC (13). Several possible genetic associations have been found in this breed (13).

Anti-seizure medication (ASM) plays a central role in the treatment of canine IE. The efficacy of registered ASMs varies. Treatment success in dogs with IE is reported in 70 to 80% with phenobarbital (14, 15), 60% with bromide (14), and 50% with imepitoin (16–18). Like in humans with epilepsy, up to 20% to 30% of all epileptic dogs do not respond favorably to registered ASMs (19) and require additional or replacement therapy with unlicensed ASMs such as levetiracetam (20, 21), gabapentin (22), zonisamide (23), or felbamate (24).

Seizure-precipitating factors (SPFs) (also termed “triggers” or “provocative factors”) have been studied and reported in both humans (25–27) and dogs with IE (28). In humans, several SPFs have been identified, such as stress, age, gender, sleep deprivation, and weather (26, 29–33). In dogs stress, hormones, sleep deprivation, and the weather have been reported as SPFs (28, 34). The reduction of stress by itself does not prevent ES but can reduce the number of seizures.

Dogs are likely the most social companions of humans, although there are breed differences. The BC is described as a very sensitive dog^1^ which has just recently been studied by comparing the Husky (an independent worker) and the BC (cooperative worker) (35). BCs have a strong focus on their owners, and this may have a clear influence on their and their owners' stress level. Importantly, veterinarians should be aware of their own impact on owners of dogs with IE (36).

In this article, we describe six unrelated BCs with IE, of which we believe that it is, in part, a genetic disorder, with several remarkable SPFs. By avoiding these SPFs, the outcome of these dogs improved dramatically. These cases illustrate that recognizing the SPF(s) and taking measures with regard to the management thereof can lead to a major reduction in ES frequency or even achieving seizure freedom.

## Dogs

The dogs described in this manuscript were all referred to one of the authors for consultation, management of IE, and/or further diagnostic testing. All six dogs are purebred BCs with a pedigree of the English Kennel Club,^2^ Dutch Kennel Club,^3^ or the International Sheep Dog Society.^4^ All owners provided footage of their dog having an ES. The videos showed signs consistent with generalized tonic–clonic seizures (GTCS) (37). The possibility of focal ES evolving to become generalized could not be excluded based on these videos. All owners participated in two earlier studies published by our group (6, 34). The clinical workup was performed using the criteria published by the International Veterinary Epilepsy Task Force (IVETF) (38). All dogs underwent a physical neurological examination, CBC, serum chemistry, fasting and post-prandial bile acids, and urine examination to exclude metabolic causes of ES (reactive seizures) [tier I level of confidence that was idiopathic (presumably genetic) epilepsy] (38). In four dogs, a tier II level of confidence was reached [an unremarkable magnetic resonance imaging (MRI) study (Canon^®^ Vantage Elan, 1.5 Tesla) of the brain]. In two of these dogs a CSF examination was performed and found unremarkable (38). All blood examinations for the first three cases were performed at the University Laboratory of the Department of Clinical Sciences, Faculty of Veterinary Medicine, Utrecht University (Utrecht, The Netherlands). The last three cases were performed at the IDEXX laboratory Europe BV (Hoofddorp, The Netherlands). All owners agreed to participate in this study and the earlier studies by informed consent.

### Case 1

A 1.5-year-old female BC was referred for consultation. The owner reported that she was a very sensitive dog and could become reactive and show aggression toward strangers in a busy environment. Recently, a cluster of five GTCS was witnessed by the owner, after which a referral was advised. At the time of referral, a tier I level of confidence for the diagnosis of IE had been reached (38). Treatment was started with potassium bromide (Libromide^®^, Dechra BV, Oudewater, The Netherlands); despite a serum level of 2,000 mg/l, she appeared unresponsive to treatment as the dog continued to seizure on a monthly basis after which levetiracetam (three times daily 23 mg/kg; Levetiracetam Aurobindo Pharma BV, Baarn, The Netherlands) was added. Despite this, the number of seizure was only reduced by 25%. Due to the side effects of the medication, incoordination, and drowsiness, the owner was forced to stop the dog's agility training. The owner observed that keeping the dog busy helped in reducing her stress level. She had learned that music therapy helped to keep the dog calm (39). Moreover, the owner started to train the dog in recognizing different objects and retrieving them. During a period of 8 years, the dog learned to retrieve over 171 different objects and became a world champion in memorizing objects.^5^ After the start of the music therapy and training, the dog became seizure free. Serum levels of bromide were above 2,000 mg/l (reference values 800–3,000 mg/l) at each yearly control visit. Several months prior to the death of the dog, the owner was forced to stop working with the dog as the dog developed a very nasty skin rash that could not be stopped despite dermatological intervention. As bromide can have dermatological side effects (40), it was stopped and replaced by phenobarbital (Phenoral^®^, AST Pharma, Oudewater, The Netherlands). However, the dermatological issues did not resolve, despite the bromide serum level had dropped to zero. Just prior to euthanasia, the dog started seizuring again. In this dog, working intensively with the dog appeared to help in getting the dog seizure free. One could say that the SPF in this dog appeared to be the lack of intellectual challenge.

### Case 2

Two male BCs, not related to each other but of the same household, were referred to as they both had developed GTCS. At the onset of ES, one dog was 1.5-year-old and the other dog was 2-year-old. At the time of referral, a tier I level of confidence for the diagnosis of IE had been reached (38). During the preceding year, 1–2 CS per month were recorded by the owners. Both dogs were already treated with phenobarbital (serum levels of 27 and 32 mg/l, respectively; Phenoral^®^), and bromide (Libromide^®^) was added. After 4 months, serum levels within the reference range for the treatment of IE were reached (1,850 and 2,200 mg/l, respectively), but both dogs were refractory to this treatment. Levetiracetam (Levetiracetam Aurobindo^®^) was added as a third medication but the reduction in frequency was limited. The owners experienced the fact that they had two dogs with refractory epilepsy as stressful. After 1 year of referral, one of the two dogs developed a status epilepticus that could not be stopped by the referring veterinarian, and the owner elected euthanasia for this dog. At a follow-up appointment of 3 months after the death of the other dog, the second dog became seizure free. This dog remained seizure free, with the prescribed medication, until his death at the age of 12 years. The possible SPF in this case was most likely the interaction with the other seizuring dog, and/or the possible stress the owners had.

### Case 3

A 1-year-old female BC was bought to be trained as an assistant dog for the family's severely autistic 12-year-old son. After its arrival, the dog started to show GTCS and CS. A tier II level of confidence for the diagnosis of IE was reached (38). Treatment was started with phenobarbital (Phenoral^®^); despite a serum level of 35 mg/l, treatment was unsuccessful, and bromide (Libromide^®^) and levetiracetam (Levetiracetam Aurobindo^®^) were added as pulse therapy (40 mg/kg, directly after an ES, to prevent or stop CS). Despite a bromide serum level of 2,200 mg/l, the dog did not respond to treatment and remained refractory. After 6 months, the owner requested euthanasia. Relocation was suggested as an alternative, as she clearly could not function as an assistant dog. The dog was kept on the medication and moved to a farm in the countryside. Up to now, 4 years after relocation, the dog is seizure free. The possible SPF in this case is most likely the interaction with the autistic son.

### Case 4

A 1-year-old male BC was bought as a family dog. The owner lives in a busy city close to the seashore. The owner crosses a busy road to walk the dog on the beach every day, multiple times a day. The dog's habit is to bark at and chase cars. On several occasions, the dog even tried to bite car tire. After a few months, the dog started to show GTCS and CS on a monthly basis, after which he was referred. A tier II level of confidence for the diagnosis of IE was reached (38). Treatment was started with phenobarbital (Phenoral^®^), and a serum level of 25 mg/l was reached. Despite a minimal reduction in ES frequency (1 ES/month), the owner elected to neither raise the dose nor add another ASM.

The owner also owns another house on one of the Dutch isles. This house is, again, situated close to the seashore. When the owner and his dog visit and live in this house, they also go for daily walks on the beach, but as this is an almost car-free island without busy roads, the dog does not need to cross a street with cars. During the lengthy periods that the family stays on this isle, the dog is seizure free. However, when they return to their city house, the dog starts seizuring again. Up to now, 2 years after referral, this remains seizures in the city but seizure-free on the island. We hypothesize that the SPF in this case is either a very busy street or maybe the owner is less stressed when he resides in the city.

### Case 5

A 2-year-old male BC was bought to help the owner recover from a traumatic accident. He developed a strong bond with his dog, and they practically lived together 24/7. When the owner returned from a brief period of 5 days of separation, the dog suffered a GTCS. A tier II level of confidence for the diagnosis of IE was reached (38). The owner elected not to start the treatment. During the following period of 2 years, the owner reported that the dog would have a GTCS during two periods he had to travel abroad for his work. However, as long as the owner stayed at home in close contact with the dog, the dog was seizure-free for nearly a year. The SPF, in this case, is the owner being not in close contact with his dog.

### Case 6

A 1-year-old male BC first suffered GTCS the day after a walk on the beach. The owner lives a few kilometers from the seashore. They walked the dog on the beach a few times per month. They noticed that if they played with a ball, the dog would suffer from GTCS the day after. A tier II level of confidence for the diagnosis of IE was reached (38). The owner thought that the ESs might be related to playing with the ball. At our request, they started walking the dog on the beach with and without throwing the ball and, likewise, did the same in the countryside. Remarkably, the dog neither had an ES the following day when they had walked in the countryside, with or without playing with a ball, nor when they had walked on the beach without throwing a ball. However, if they throw a ball on the beach, the dog will suffer from GTCS the day after. During a period of several months, this was evaluated, and the outcome remained the same. The SPF is the throwing of the ball at the beach.

## Discussion

We encountered six BCs suffering from IE in which remarkable observations were made regarding SPFs. The first dog became seizure-free once the owner started with music therapy and a learning game. The dog had been unresponsive to the chosen treatment and apparently only became seizure-free after the start of music therapy and gaming. When the owner was forced to stop the learning game, the dog had a seizure again and replaced the bromide with phenobarbital (14, 41). It is possible that the phenobarbital is, in this dog, less effective than the potassium bromide, but this is in contrast to what has been published earlier (14, 41). It appears that this dog only became seizure-free after she started with the game of object retrieval. It can be coincidental, but only when the owner stopped the game of object retrieval, the dog started, after 8 years of being seizure-free, to seizure again.

In case number 2, the surviving dog stopped seizuring once the other dog had died. It is unlikely that the surviving dog was suffering from psychogenic non-epileptic seizures (PNES) (42), as this has not, to the best of authors' knowledge, been described in dogs. PNES are considered to be personality pathology, psychiatric illness, and experiential and behavioral manifestations of depression (42). Moreover, we hypothesize that the relation between these two dogs, or the possible stress this situation has caused for the owners, caused such a level of stress that both dogs became refractory. Chronic stress has been described as a possible SPF in humans (43, 44).

The third case illustrates most likely the sensitivity of the BC breed. We concluded after our work-up that this dog had an idiopathic (presumably genetic) epilepsy. Hence, the dog was genetically prone to develop epilepsy, but the environment of the dog was the SPF to show the seizures. Again, this is unlikely to be a dog with PNES. After relocation, this dog became seizure-free and behaved normally. It is possible that the new owner missed a single seizure, but as the dog was suffering from CS, it is not very likely that they missed complete clusters.

In case 4, it is possible that the stress the dog encounters in the busy city is an SPF which is absent on the island. It is also possible that the owner plays a role. The owner may be more relaxed on the island. However, whatever the SPF is, the result was remarkable. Both cases 4 and 5 kept on having seizures, but in case 4, it appeared to have a relation with the domestic environment, and in case 5 with the work of the owner. In the latter case, there seems to be a direct relation between the level of owner interaction and seizures in his dog. In case 6, it was not just the act of throwing and playing with a ball but rather where the owners would throw the ball. In cases 4, 5, and 6, the owners tested these relationships repeatedly and found consistent results, which seem to contradict that these observations are mere chance.

Stress as an SPF has been described in several studies addressing epilepsy in humans (25–27, 30, 32). The number of studies on dogs is limited. Stress was identified as one of the SPFs in a small number of dogs, according to Forsgård et al. (28), and our group identified stress as a factor in a study with 116 BCs (34). Moreover, most likely, there are breed differences as well. In a recent study, we observed a clear breed (6). We hypothesize that although epilepsy in many breeds is most likely of genetic origin (7), there are striking differences in possible SPFs for the different breeds. Dogs, that are judged by the breeders and breed clubs as more sensitive in nature, like the BC, Australian Shepherd and Belgian Shepherd, may be more susceptible to SPFs as described than less sensitive breeds such as the Dutch Partridge dog, Labrador retriever and Golden retriever. This might even have implications for genetic studies as phenotyping may be more difficult.

Just recently, it was observed that the relationship between the owner and veterinary surgeon plays a role in the management of dogs with IE (36). These six cases illustrate that we also need to take into account the domestic situation of the dog. There is an influence of both the owner and the environment. The owner or environment does not cause epilepsy but maybe (part of) an SPF.

The limitation of this study is the mere fact that it is a very small group of dogs. However, they are all purebred BCs, and in all six dogs, we identified a possible SPF.

## Conclusion

Veterinarians treating IE in dogs need to consider breed, their own relation with the owner, and the domestic situation or environment in which the dog is housed. Preferably larger prospective studies are needed to further investigate the nature and role of SPFs in dogs with epilepsy.

## Data availability statement

The raw data supporting the conclusions of this article will be made available by the authors, without undue reservation.

## Ethics statement

Ethical approval was not required for the studies involving animals in accordance with the local legislation and institutional requirements because the study describes six referred patients. Written informed consent was obtained from the owners for the participation of their animals in this study.

## Author contributions

PM: Conceptualization, Data curation, Formal analysis, Funding acquisition, Investigation, Methodology, Project administration, Supervision, Validation, Writing–original draft, Writing–review and editing. KS: Data curation, Investigation, Validation, Writing–review and editing.

## Footnotes

^1^ http://bordercolliehelp.com/border-collies-are-sensitive-dogs

^2^ https://www.thekennelclub.org.uk/

^3^ https://www.houdenvanhonden.nl/

^4^ https://www.isds.org.uk/

^5^ https://www.youtube.com/watch?v=UBakHiQ-0SE
